# Deposition, Characterization, and Modeling of Scandium-Doped Aluminum Nitride Thin Film for Piezoelectric Devices

**DOI:** 10.3390/ma14216437

**Published:** 2021-10-27

**Authors:** Qiaozhen Zhang, Mingzhu Chen, Huiling Liu, Xiangyong Zhao, Xiaomei Qin, Feifei Wang, Yanxue Tang, Keat Hoe Yeoh, Khian-Hooi Chew, Xiaojuan Sun

**Affiliations:** 1 Shanghai Normal University, Shanghai 200234, China; chenmz_0126@163.com (M.C.); 1000478898@smail.shnu.edu.cn (H.L.); xmqin@shnu.edu.cn (X.Q.); f_f_w@sohu.com (F.W.); yanxuetang@shnu.edu.cn (Y.T.); 2 Department of Electrical and Electronic Engineering, Lee Kong Chian Faculty of Engineering and Science, Universiti Tunku Abdul Rahman, Kajang 43000, Malaysia; keathoe.yeoh@gmail.com; 3 Department of Physics, University of Malaya, Kuala Lumpur 50603, Malaysia; 4 State Key Laboratory of Luminescence and Applications, Changchun Institute of Optics, Mechanics and Physics, Chinese Academy of Sciences, Changchun 130033, China; sunxj@ciomp.ac.cn

**Keywords:** piezoelectric thin film, scandium-doped aluminum nitride, crystal structure, first-principles calculation

## Abstract

In this work, we systematically studied the deposition, characterization, and crystal structure modeling of ScAlN thin film. Measurements of the piezoelectric device’s relevant material properties, such as crystal structure, crystallographic orientation, and piezoelectric response, were performed to characterize the Sc_0.29_Al_0.71_N thin film grown using pulsed DC magnetron sputtering. Crystal structure modeling of the ScAlN thin film is proposed and validated, and the structure–property relations are discussed. The investigation results indicated that the sputtered thin film using seed layer technique had a good crystalline quality and a clear grain boundary. In addition, the effective piezoelectric coefficient *d*_33_ was up to 12.6 pC/N, and there was no wurtzite-to-rocksalt phase transition under high pressure. These good features demonstrated that the sputtered ScAlN is promising for application in high-coupling piezoelectric devices with high-pressure stability.

## 1. Introduction

Piezoelectric devices have received increasing interest in a variety of applications in advanced electronic and in-formation industries, where they are used as resonators, filters, sensors, and actuators [1,2,3,4,5]. The properties of those piezoelectric devices mainly depend on the choice of piezoelectric materials. Bulk crystal materials are the most commonly used, but piezoelectric thin films such as zinc oxide (ZnO) and aluminum nitride (AlN) are emerging alternatives. Recently, AlN has attracted much attention due to its outstanding features such as high thermal stability, high acoustic velocity, low acoustic loss, and in particular, good compatibility with the complementary metal–oxide–semiconductor (CMOS) manufacturing process, which is promising for integrated sensors/actuators on silicon substrates. As for piezoelectric device applications, piezoelectricity is the main possibility investigated to offer efficiency electromechanical coupling. However, the piezoelectric response of pure AlN thin film is relatively small (*d*_33_ ≈ 5.5 pC/N) [6], which results in a low electromechanical coupling coefficient (*k*_t_^2^ = 6~7%) [7], and thus limits its important applications in technology such as high-sensitivity micromachined medical ultrasonic devices and wideband wireless communication filters [8,9].

It is known that the IIIA nitrides are AlN, GaN, and InN, and that these nitrides have a wurtzite structure [10,11]. First-principle calculations have indicated that a ScN wurtzite structure and the fabrication of Sc-IIIA-N nitrides are possible [10,12], and researchers found that the piezoelectric response of hexagonal Sc-IIIA-N was enhanced [13,14,15]. To enhance the piezoelectricity of AlN, Akiyama et al. [6] first fabricated and investigated piezoelectric properties of scandium (Sc)-doped AlN; i.e., the Sc_x_Al_1−x_N alloy. It was demonstrated that the Sc_x_Al_1−x_N films with a Sc concentration of 43% exhibited a four times larger piezoelectric response than pure AlN films. Wingqvist et al. [7] validated that the electromechanical coupling coefficient *k*_t_^2^ of Sc_0.3_Al_0.7_N could be enhanced up to 15%, almost twice that of pure AlN (7%). During the last decades, Sc_x_Al_1−x_N thin film layered piezoelectric structures achieving strong coupling have attracted increasing attention worldwide. The Sc_x_Al_1−x_N thin-film-based resonators with high Sc concentration offer prospects for developing high-frequency and broad wideband acoustic wave filters for fifth-generation (5G) mobile communication [16,17,18]. Nevertheless, mass production of such ScAlN films (more than 20% Sc content) with good crystalline quality and excellent piezoelectric properties is still difficult, and thus gives rise to limitations in wide applications [19,20,21,22]. To deal with this problem, the crystal structure of ScxAl1-xN thin film is worthy of being explored in great detail. Previously, Akiyama et al. reported XRD patterns and lattice constants of Sc_x_Al_1−x_N alloys at various different Sc concentrations [6]. Zukauskaite et al. presented TEM micrographs and corresponding SAED patterns of AlN, Sc_0.2_Al_0.8_N, and Sc_0.3_Al_0.7_N films, and studied the microstructure and crystal quality of the films [23]. Deng et al. reported Raman scattering spectra for a sapphire substrate and Sc_x_Al_1−x_N layers with x = 0–0.16 [24]. However, these previous studies mainly focused on the influences of Sc concentration on piezoelectric properties, so there is still a lack of information from a systematic investigation. For example, there is no report on the structure properties of Sc_x_Al_1−x_N alloy thin films under high pressure or a high electric field, and it is very important to disclose the coupling between elastic and electric properties and structure–property relations, especially for piezoelectric-susceptible materials.

Therefore, in this work, a ScAlN thin film was systematically investigated in terms of deposition, characterization, and crystal structure modeling. First, the Sc_0.29_Al_0.71_N thin film was deposited on a 6-inch Mo/SiO_2_/AlN/SOI substrate by employing a pulsed DC magnetron sputtering system. Then, measurements of the piezoelectric-device-relevant material properties, such as crystal structure, crystallographic orientation, and piezoelectric response, were performed to characterize the sputtered thin film. The crystal structure and lattice patterns of the sputtered thin film were investigated by high-resolution transmission electron microscopy. According to the analysis of the selected area electron diffraction pattern, the crystal structure of Sc_0.29_Al_0.71_N was hexagonal phase. First-principles calculations were also performed to study the structural and electronic properties of Sc_0.29_Al_0.71_N. The calculated lattice parameters were in good agreement with the measured results. The chemical bonding states of Sc-doped AlN were investigated by X-ray photoemission spectroscopy. In addition, high-pressure Raman spectroscopy was employed to study the evolution of vibrational frequencies of the ScAlN phonons. The investigation results indicated that the ScAlN thin film could maintain material properties under high pressure, which is very important to ensure stable and reliable device performance, especially for piezoelectric pressure sensors.

## 2. Experimental

### 2.1. Deposition of ScAlN Thin Film

In this work, a conventional pulsed DC magnetron sputtering system (Sigma fxP PVD system, SPTS) was employed for the ScAlN thin-film deposition. This PVD cluster system consisted of four process chambers (AlN chamber, AlScN chamber, Mo chamber, and preclean chamber) and one transport chamber. The AlScN sputtering chamber was equipped with a 12-inch Sc_0.3_-Al_0.7_ alloy target. The ScAlN film was deposited on 6-inch Mo/SiO_2_/AlN/SOI substrate without vacuum breaking. Table 1 shows the deposition conditions of the ScAlN deposition. For deposition, the SOI wafer was cleaned successively by high temperature degas and argon ion soft etching in the clean chamber to ensure clean surfaces for film growth, and a Mo (110) thin film was prepared as a bottom electrode for electrical property measurements. During the deposition, the substrate was rotated 90° four times to ensure film uniformity. It is worth mentioning that before sputtering the Mo layer, we used SiO_2_ and AlN as a seed layer to improve the quality of the ScAlN (002) with better crystal orientation. 

### 2.2. Characterization of ScAlN Thin Film

Next, measurements of the piezoelectric-device-relevant material properties were performed to characterize the Sc_0.29_Al_0.71_N thin film grown using pulsed DC magnetron sputtering. A scanning electron microscope (SEM, S-4800, Hitachi, Chiyoda, Japan) was used to investigate the microstructure and obtain a cross-sectional view of the sputtered film, and the component analysis was performed with an energy dispersive spectroscope (EDS). Transmission electron microscopy (TEM, JEM-2100F, JEOL, Akishima, Japan) was used to characterize the microstructure at the nanometric level. The crystal orientations and piezoelectric properties of the Sc_0.29_Al_0.71_N thin films were characterized by high-resolution X-ray diffraction (HRXRD, D8 ADVANCE, BRUKER, Billerica, USA) and a ferroelectric analyzer (TF-2000, aixACCT, Aachen, Germany), respectively.

In order to investigate the pressure-induced phase transformations, in situ high-pressure Raman measurements (up to 20 GPa) were conducted in a symmetric diamond anvil cell (DAC) with a diamond culet size of 300 μm in diameter. A small piece of the Sc_0.29_Al_0.71_N sample with ~27 μm thickness on the Mo/SiO_2_/SOI substrate was loaded into a sample chamber 100 μm in diameter drilled in the center of a T301 stainless-steel gasket. Silicone oil was used as the pressure-transmitting medium, and the pressure calibration was done using ruby fluorescence. An argon ion laser (=532 nm) was used as the excitation source, and the diameter of the focused laser radiation area was 10 μm.

### 2.3. Crystal Structure Modeling of ScAlN Thin Film

As for modeling of crystal structure of the sputtered Sc_0.29_Al_0.71_N thin film, first-principles calculations based on density functional theory (DFT) were carried out using the Quantum ESPRESSO codes [25,26]. PBEsol functional was used within the generalized gradient approximation (GGA) [27]. We employed the PAW pseudopotential to describe the electron–ion interaction. The plane-waves kinetic energy and charge densities cutoff were set to 50 Ry and 402 Ry, respectively. All the calculations were carried out on a 5 × 5 × 5 Monkhorst–Pack grid. Structural relaxation was carried out until the residual force had converged to less than 0.0001 Ry/a.u. 

The calculated crystal structure of the Sc_0.29_Al_0.71_N was verified by comparison of the lattice constant to the TEM measurement results at the micro-nanometric level. In addition, X-ray photoelectron spectra (XPS) was used to characterize the chemical bonds of the Sc-doped AlN thin film. The X-ray photoemission spectroscopy (XPS) measurements were carried out on a VG ESCALAB MKII spectrometer (VG Scientific Ltd., London, UK) using a monochromatic Al *Kα* X-ray beam

## 3. Results and Discussion

### 3.1. Microstructural and Crystal Structure Properties 

**Figure 1:**
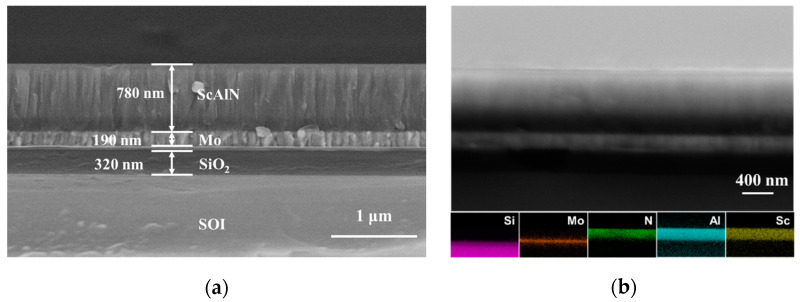
SEM micrographs and analysis of ScAlN thin film deposited on Mo/SiO_2_/AlN/SOI: (**a**) SEM micrographs; (**b**) EDS mapping.

Figure 1a shows the SEM cross-sectional view of the microstructure of the ScAlN thin film deposited on the Mo/SiO_2_/SOI substrate. The SEM image indicates that the prepared thin film had a visible columnar structure that grew perpendicular to the substrate. This columnar-like growth was similar to the columnar microstructure of pure AlN [28]. It can be observed that the SAlN thin film had a good crystalline quality and a clear grain boundary. The ScAlN thin film thickness was about 780 nm, and the thickness was about 190 and 320 nm for the Mo and SiO_2_, respectively. Figure 1b shows EDS mapping of the microstructure in a cross-sectional view. It shows that the prepared ScAlN film substrate had a layered structure; namely, ScAlN/Mo/SiO_2_/SOI, and the elements of Sc, Al, and N were evenly distributed in the ScAlN film. Through the EDS analysis, the atomic ratio for Sc:Al:N was found to be 0.29:0.71:1, which fit fairly well with the concentration of the Sc_0.3_-Al_0.7_ alloy target. This provided clear visual evidence that Al, N, and Sc elements were homogeneously distributed in the ScAlN. 

**Figure 2:**
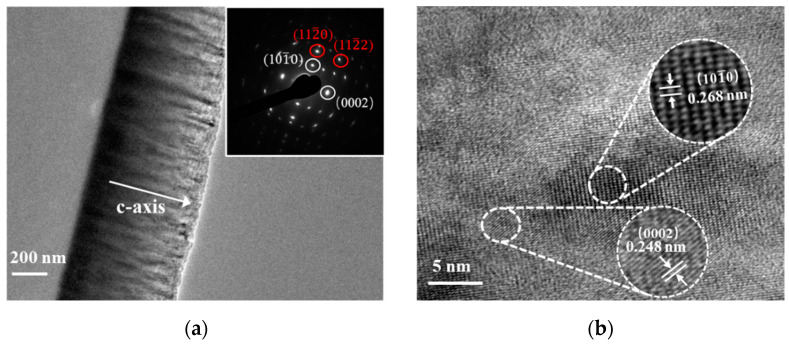
TEM images of the prepared Sc_0.29_Al_0.71_N thin film: (**a**) cross-sectional TEM image and selective area electron diffraction pattern (SAED); (**b**) high-resolution TEM image.

Next, we turned to the detailed characterization of the crystal structure at the micro-nanometric level. Figure 2 illustrates the TEM plane views of the prepared Sc_0.29_Al_0.71_N thin film. It can be seen in Figure 2a, the cross-sectional TEM image, that the Sc_0.29_Al_0.71_N thin film had a uniform columnar structure and showed c-axis texture. The selective area electron diffraction (SAED) pattern (shown in the inset of Figure 2a) was composed of discrete spots in an arclike arrangement. Meanwhile, a slight spot broadening in the circumferential direction was present, which indicated that the crystalline quality of Sc_0.29_Al_0.71_N was not as good as the pure AlN thin film. It was noted that the crystal structure of Sc_0.29_Al_0.71_N was also found to have a hexagonal structure from the diffraction spots of the SAED pattern, and additional reflections such as (10$\bar{1}$0) and (11$\bar{2}$0) also appeared due to stacking faults. The HRTEM image of the same Sc_0.29_Al_0.71_N thin film (shown in Figure 2b) revealed that the crystal distortion and stacking faults occurred with the addition of Sc. The lattice plane spacing of the (0002) plane was about 0.248 nm, which was slightly smaller than that of the AlN (0.249 nm). A small amount of twinning with orientation (10$\bar{1}$0) was also visible, as the lattice parameters of (0002) and (10$\bar{1}$0) were very close to each other. These results indicated that the prepared Sc_0.29_Al_0.71_N thin film was polycrystalline.

**Figure 3:**
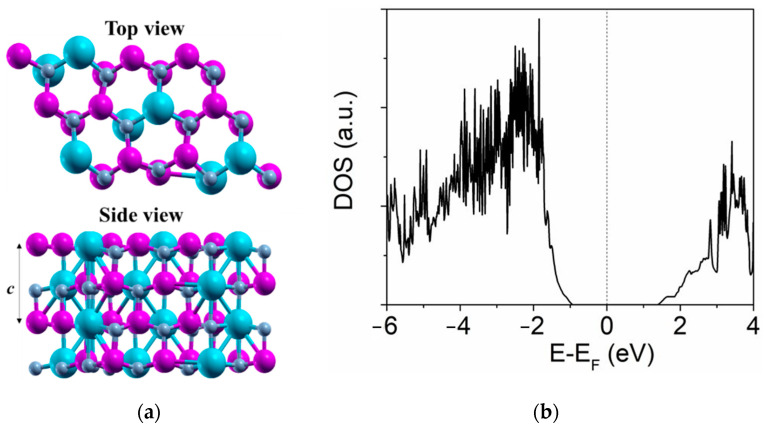
(**a**) Top and side views of the calculated crystal structure; and (**b**) density of states (DOS) for the Sc_0.29_Al_0.71_N crystal. The purple, grey, and bluish spheres denote Al, N, and Sc atoms, respectively.

The interplanar spacing of the hexagonal system is given by:

(1) $$\frac{1}{d^{2}}=\frac{4}{3}\left(\frac{h^{2}+hk+k^{2}}{a^{2}}\right)+\frac{l^{2}}{c^{2}},$$

where *h*, *k,* and *l* are indices of the crystal plane; and *d* is the interplanar spacing. According to the obtained parameters shown in Figure 2, the *a* lattice constant and *c* lattice constant of the Sc_0.29_Al_0.71_N could be estimated as 3.0997 Å and 4.9569 Å, respectively. In order to further validate our results, we performed first-principles calculations on the structural properties of Sc_0.29_Al_0.71_N. Figure 3a shows the predicted crystal structure of the Sc_0.29_Al_0.71_N alloy. From the density of states (DOS) analysis shown in Figure 3b, we found that the Sc_0.29_Al_0.71_N remained semiconducting. The lattice parameter *a* in a pristine AlN crystal is defined as the distance between the N-N or Al-Al atoms within a hexagon ring. However, when the AlN was doped with Sc, the value of lattice parameter *a* varied due to localized strain. As shown in Table 2, in this work, the calculated value of *a* was taken as the average of all the N-N, Al-Al, Al-Sc, and N-Sc distances within the hexagon ring in a Sc_0.29_Al_0.71_N unit cell. The calculated lattice parameters of the crystal structure were *a* = 3.2619 Å and *c* = 4.9633 Å. These values were in good agreement with those previously reported Akiyama’s work, which were calculated using images of electron-beam diffractions [6]. 

**Figure 4:**
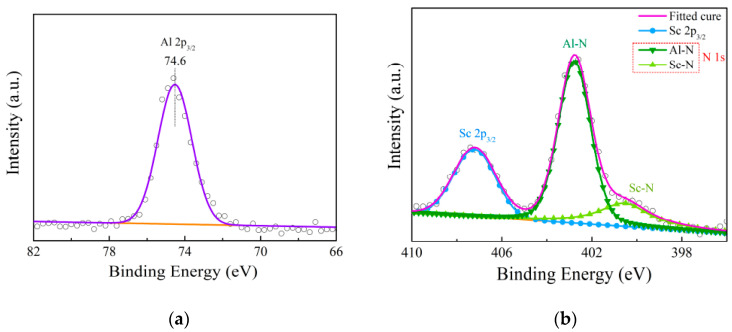
XPS elemental spectra for Sc_0.29_Al_0.71_N thin film: (**a**) Al 2p_3/2_; (**b**) N 1s and Sc 2p_3/2_.

Furthermore, X-ray photoelectron spectroscopy (XPS) was used to characterize the chemical bonds of the Sc-doped AlN thin film. Note that all XPS data were calibrated with 284.8 eV of C 1s peak. Figure 4a shows the Al 2p_3/2_ peak of the Sc_0.29_Al_0.71_N. The spectra exhibited only one intense peak related to aluminum, indicating that Al was the metal species with no inherent oxide. In Figure 4b, the nitrogen peak consists of two subpeaks of binding energies of 400.3 eV and 402.8 eV. The bigger subpeak at 402.8 eV was one belonging to the Al-N bond, and the smaller one was ascribed to the Sc-N bond. It can be seen that scandium had only one obvious peak in the Sc_0.29_Al_0.71_N thin film, indicating that there was only one way of binding Sc atoms. Through the analysis of the XPS, the data provided strong evidence that the Sc element formed a Sc-N combination in the Sc_0.29_Al_0.71_N thin film. It provided an effective basis for establishing a crystal structure model.

### 3.2. Crystal Orientation and Piezoelectric Properties

**Figure 5:**
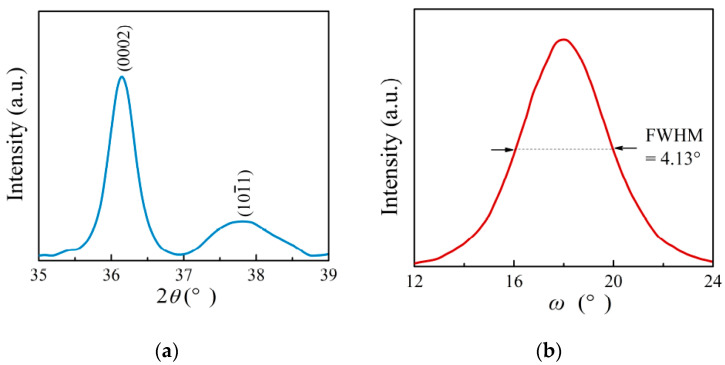
(**a**) High-resolution XRD pattern and (**b**) X-ray rocking curve of the Sc_0.29_Al_0.71_N thin film.

The crystal orientation of the Sc_0.29_Al_0.71_N thin film was investigated by HRXRD. The HRXRD measurements were carried out using the Cu *Kα1* line (1.54056 Å). Figure 5a shows the HRXRD spectrum in *θ*–2*θ* scan mode of the Sc_0.29_Al_0.71_N thin film. There were two peaks of (0002) and (10$\bar{1}$1) in the 35–39° scanning range. In comparison, it showed a strong (0002) preferred orientation. Then, the crystallinity of thin films was investigated by XRD rocking-curve measurement. The full width at half maximum (FWHM) of the X-ray rocking curve is shown in Figure 5b. The FWHM value of the (0002) peak in the Sc_0.29_Al_0.71_N thin film was 4.13°. Although the crystal orientation was not high compared to that of single-crystal AlN film [29], this FWHM value suggested a strongly c-axis-oriented polycrystalline structure of the Sc_0.29_Al_0.71_N thin film.

**Figure 6:**
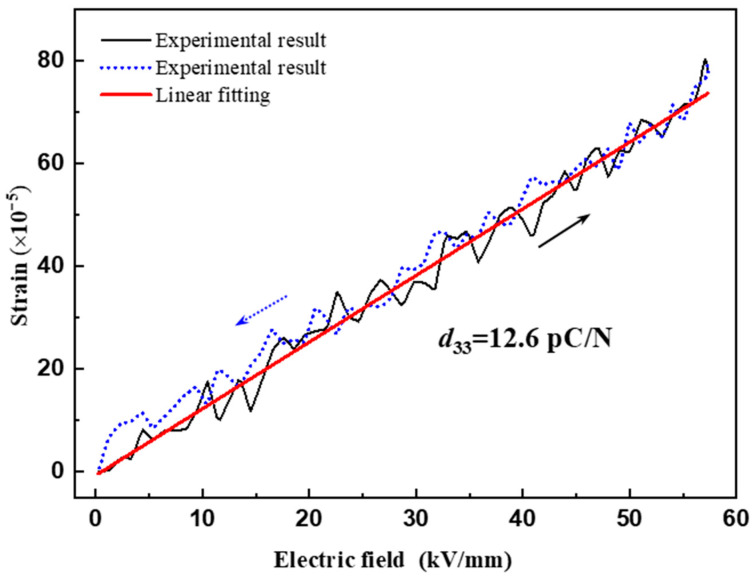
The measured field-induced strain of the Sc_0.29_Al_0.71_N thin film as a function of an applied electric field.

Next, the ferroelectric hysteresis and field-induced strain curve of the Sc_0.29_Al_0.71_N thin film was investigated with a ferroelectric analyzer (Aixacct TF-2000). Figure 6 shows the measured field induced strain of the Sc_0.29_Al_0.71_N thin film as a function of an applied electric field. The experimental data showed that the induced strain varied linearly with both an increasing and decreasing electric field. An effective piezoelectric coefficient *d*_33_ of 12.6 pC/N could be estimated by linear fitting, which was slightly small compared to that of Akiyama’s work (~13.7 pC/N) [30]. This may have been caused by the difference in thin film quality or the different measurement tool. It is worth noting that the obtained *d*_33_ for Sc_0.29_Al_0.71_N thin film was almost 2.2 times larger than that of the AlN film. Such behaviors allowed us to know that it could be used in piezoelectric devices supporting strong electromechanical coupling. Taking an example of a surface acoustic wave resonator using the AlN film layered structure, in our previous work, the authors demonstrated that the effective coupling factor *K*^2^ was dramatically enhanced from 1.45% up to 10.5% by replacing AlN with ScAlN [31].

### 3.3. High-Pressure Properties

**Figure 7:**
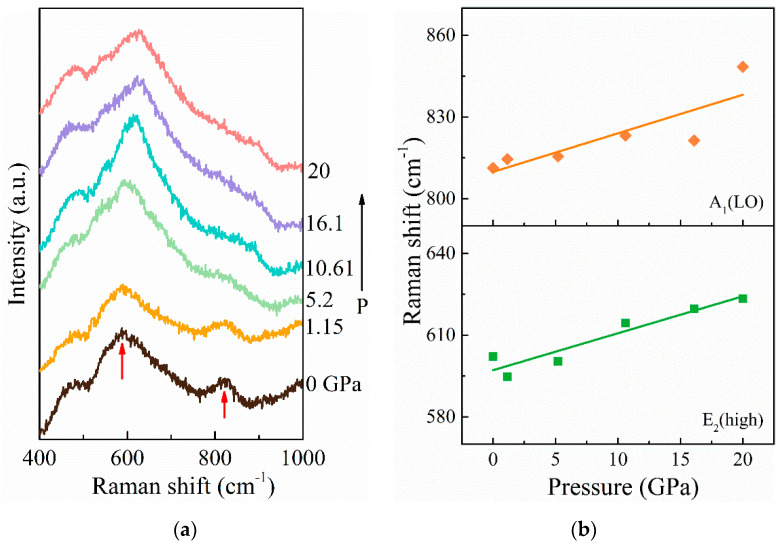
(**a**) Pressure evolution of Raman spectra of the Sc_0.29_Al_0.71_N thin film for pressurization cycle; (**b**) pressure dependence of E_2_(high) and A_1_(LO) phonon modes.

It is known that wurtzite-to-rocksalt phase transitions are typically observed in AlN at pressures of around 20 GPa [32]; nevertheless, ScAlN should be varied when doped with Sc. Hence, Raman measurements were taken of the Sc_0.29_Al_0.71_N thin film for analyses of its high-pressure properties. Figure 7a presents pressure-dependent Raman spectra of the Sc_0.29_Al_0.71_N thin film. The Raman spectra contained two peaks, at ~600 and ~810 cm^−1^, corresponding to the E_2_(high) and A_1_(LO) phonon modes, respectively [24,33]. It was found that the Raman bands of the wurtzite phase in AlN weakened above 18 GPa and disappeared at about 20 GPa due to the phase transition to the rocksalt structure [32,34,35]. However, it can be seen in Figure 7a that Raman bands in the Sc_0.29_Al_0.71_N thin film shifted continuously to higher phonon energy. No broadening or intensity loss of the E_2_(high) phonon mode was observed. The spectra were fitted with the Lorentz functions to determine the phonon wavenumber. The fitted results shown in Figure 7b were the measured frequencies for E_2_(high) and A_1_(LO) modes as a function of pressure, while the lines were obtained from linear fitting. With increasing pressure, the spectral deconvolution of the Raman spectra revealed a slightly linear enhancement in the frequency of phonon modes, and the decrease in lattice constants was related to the increase in phonon frequencies. Compared with the pressure dependence of the phonon frequencies of the Raman-active modes in wurtzite AlN, the rate of variation of frequencies with pressure for the E_2_(high) mode in Sc_0.29_Al_0.71_N was smaller. There was no wurtzite-to-rocksalt phase transition under high pressure (≤20 GPa). This means that piezoelectric devices using Sc_0.29_Al_0.71_N thin film could maintain material properties under high pressure, which is very important to ensure stable and reliable device performance, especially for piezoelectric pressure sensors.

## 4. Conclusions

In this work, a Sc_0.29_Al_0.71_N piezoelectric thin film measuring 780 nm thick was prepared with a conventional pulsed DC magnetron sputtering system on a Mo/SiO_2_/AlN/SOI substrate. Characterization of the microstructural and crystal structure properties for the sputtered Sc_0.29_Al_0.71_N thin film were performed. The SEM micrographs showed that the Sc_0.29_Al_0.71_N thin film had a good crystalline quality and a clear grain boundary. The TEM images revealed that crystal distortion and stacking faults occurred with the addition of Sc. The analyses of the XPS showed that the Sc element formed a Sc-N combination in the Sc_0.29_Al_0.71_N thin film. Analyses based on the DOS indicated that the Sc_0.29_Al_0.71_N remained semiconducting. The crystal structure was hexagonal phase, and the measured lattice constants *a* and *c* of Sc_0.29_Al_0.71_N were estimated as 3.0997 Å and 4.9569 Å, respectively. First-principles calculations were performed to predict the structural and electronic properties of the Sc_0.29_Al_0.71_N. The calculated lattice parameters were in good agreement with the measured results. This provided an effective basis for establishing a crystal structure model of Sc_x_Al_1−x_N for various Sc content.

Furthermore, the piezoelectric-device-relevant material properties in terms of crystal orientation and piezoelectric response, as well as high-pressure properties, were also investigated. The results demonstrated that the prepared ScAlN thin film offered high-quality crystal orientation and a high effective piezoelectric coefficient *d*_33_ of 12.6 pC/N. In addition, there was no wurtzite-to-rocksalt phase transition under high pressure (≤20 GPa), which is quite beneficial for application in strong coupling piezoelectric devices with high-pressure stability.

## Figures and Tables

**Table 1:** The deposition conditions.

Parameter	Value
Target-substrate distance	70 mm (fixed)
Substrate temperature	300 °C
Power	7500 W
RF Bias	60W
Total gas pressure	2.6 mTorr
Gas composition ratio	Ar/N_2_ = 1/3
Sputtering time	20 min

**Table 2:** The calculated lattice constants within a unit cell of Sc_0.29_Al_0.71_N.

Lattice Constant, a (Å)					
3.4792	3.1356	3.1616	3.1578	3.5957	3.0657
3.2237	3.1889	3.2135	3.3515	3.1551	3.2530
3.1376	3.5382	3.1546	3.1498	3.1403	3.5532
3.1953	3.3510	3.2578	3.1545	3.2388	3.2537
3.1257	3.1010	3.4524	3.5965	3.0894	3.1694
3.3465	3.2212	3.2344	3.1632	3.3268	3.2614
3.1820	3.1744	3.4380	3.1018	3.5349	3.1677
3.2387	3.2443	3.3773	3.2424	3.3673	3.1387
3.5242	3.0646	3.2714	3.1521	3.0802	3.2563
3.2432	3.3938	3.1559	3.3191	3.2401	3.1299
3.1781	3.5284	3.2295	3.5410	3.1566	3.1619
3.3612	3.1769	3.2690	3.2273	3.1831	3.2497
3.2599	3.1557	3.3734	3.3310	3.2445	3.2025
3.6009	3.4151	3.1811	3.2286	3.1419	3.4969
3.0974	3.1456	3.3084	3.2209	3.4348	3.1904

Note: The average and standard deviations of the calculated lattice constant a were 3.2619 Å and 0.1382 Å, respectively.

## Data Availability

Data sharing is not applicable for this article.
